# Effects of Inhaled Corticosteroids and Particle Size on Risk of Obstructive Sleep Apnea: A Large Retrospective Cohort Study

**DOI:** 10.3390/ijerph17197287

**Published:** 2020-10-06

**Authors:** Maria Paula Henao, Jennifer L. Kraschnewski, Matthew D. Bolton, Faoud Ishmael, Timothy Craig

**Affiliations:** 1 Division of Allergy, Asthma, and Immunology, Penn State Hershey Medical Center, Hershey, PA 17033, USA; faoudishmael@gmail.com (F.I.); tcraig@pennstatehealth.psu.edu (T.C.); 2 Department of Medicine, Penn State Hershey Medical Center, Hershey, PA 17033, USA; jkraschnewski@pennstatehealth.psu.edu; 3 Bioinformatics and Enterprise Information Management, Penn State Hershey Medical Center, Hershey, PA 17033, USA; mbolton@pennstatehealth.psu.edu

**Keywords:** inhaled corticosteroids, asthma, obstructive sleep apnea, particle size of inhaled corticosteroids, mass median aerodynamic diameter, localized pharyngeal side effects, pharyngeal muscles

## Abstract

*Background:* Inhaled corticosteroids (ICS) produce local effects on upper airway dilators that could increase the risk of developing obstructive sleep apnea (OSA). Given that the particle size of ICS changes their distribution, the particle size of ICS may impact the risk of developing OSA. *Objectives:* In this large retrospective study, we explore the relationship of ICS use and OSA in patients with asthma. In addition, we seek to determine if this relationship is affected by the particle size of ICS. *Methods*: Using electronic health records, we established a cohort of 29,816 asthmatics aged 12 and older with a diagnosis of asthma documented by ICD-9 or ICD-10 codes between January 2011 and August 2016. We performed analyses of variance and multivariate logistic regression analysis to determine the effects ICS on the diagnosis of OSA with sub-analysis by particle size of ICS. *Results:* Uncontrolled asthmatics showed increased odds of receiving a diagnosis of OSA whether when looking at ACT scores (adjusted odds ratio (aOR) 1.60, 95% CI 1.32–1.94) or PFT results (aOR 1.45, 95% CI 1.19–1.77). Users of ICS also had increased odds of OSA independent of asthma control (aOR 1.58, 95% CI 1.47–1.70). Notably, users of extra-fine particle ICS did not have significantly increased odds of having OSA compared to non-users of ICS (aOR 1.11, 95% CI 0.78–1.58). *Conclusions*: Use of ICS appears to be an independent risk factor for OSA. Notably, extra-fine particle size ICS do not appear to be associated with an increased risk of OSA.

## 1. Introduction

Although asthma has been associated with sleep disturbances and the development of obstructive sleep apnea (OSA), the mechanisms that underlie the relationship and the optimal approaches to subsequent therapy have not been well described [1,2,3,4,5,6,7]. Asthma is increasingly understood to be a heterogeneous and complex disease; in addition, it is believed that there may be a bidirectional relationship, such that asthma increases the risk for OSA and OSA worsens asthma symptom control [8,9]. 

Inhaled corticosteroids (ICS) are known to increase the risk for dysphonia by causing myopathy of the vocal cord adductors [10]. In the same way, ICS may produce similar local effects on the upper airway dilators that could increase the risk of developing OSA. This mechanism is hypothesized to result from raising the surrounding tissue pressure from centripetal fat accumulation and redistribution, and by diminishing the contractile properties of its protective dilator muscles. In 2009, Teodorescu et al. performed a retrospective study of 244 patients and found that ICS use increases OSA in a dose-dependent fashion [6]. Subsequently, a recent randomized control trial found a positive association between asthma and the development of OSA, indicating that OSA symptoms are more prevalent and associated with higher disease burden among severe asthmatics [8,11].

Additionally, the effect of ICS on the risk of OSA may change depending on the particle size of the ICS. Particles emitted from inhaler devices are not of uniform size and may range from <1 to >10 µm in diameter [12,13,14]. Two primary measures may be used to measure particle size of ICS: mass median aerodynamic diameter (MMAD) and fine particle fraction (FPP). Both measures are based on in vitro assessments of particle distribution by the eight-stage Andersen Cascade Impactor [14,15]. Moreover, the diameter of particles is not consistent in any given dose and may fluctuate depending on certain conditions, such as temperature, relative humidity, and inhalation flow rates [14]. To reach the airways, particles should be smaller than 5 µm; ICS with particles on the larger side of this range have more deposition in the upper airway than ICS of extra-fine particle size (<2 µm), which distribute more uniformly across the lower airway [13,14,16]. This effect may impact the risk of the development of OSA. In this large retrospective study, we examined the relationship between the use of ICS and a diagnosis of OSA. In particular, we explored the effect of particle size of different ICS on this relationship. To our knowledge, the latter relationship has not been previously evaluated.

## 2. Methods

Using electronic health records through the Penn State Clinical and Translational Science Institute and after receiving IRB approval of Human Subjects Protections Office at the Penn State Hershey Medical Center (Study ID: 00002601), we established an initial cohort of 29,816 patients with asthma over age 12. All patients had received a diagnosis of asthma by ICD-9 or ICD-10 codes between January 2011 and August 2016.

We investigated the effect of ICS use on the diagnosis of OSA with sub-analysis by the particle size of ICS. A diagnosis of OSA was determined by ICD-9 or ICD-10 codes reported during the study period. We divided the initial cohort into unique groups for separate analyses depending on the availability of data regarding asthma control. To assess the level of asthma control, we used either the asthma control test (ACT) (*n* = 4668) or pulmonary function tests (PFTs) (*n* = 2428). The ACT is a validated questionnaire that provides a numerical score to help determine asthma symptom control in patients 12 years and older [17,18]. When multiple ACT scores were available, we used the mean value. We established categories of asthma control with an ACT score of less than 20 indicating uncontrolled asthma [17,18]. Similarly, when PFT values were available, we used the mean value of forced expiratory volume in 1 second (FEV_1_) percent predicted values, and considered scores of 80 or less uncontrolled [19,20,21,22]. When possible, we adjusted our analysis for several covariates that may influence risk of OSA, including sex, race, BMI, tobacco use, and asthma control [18].

**Figure 1:**
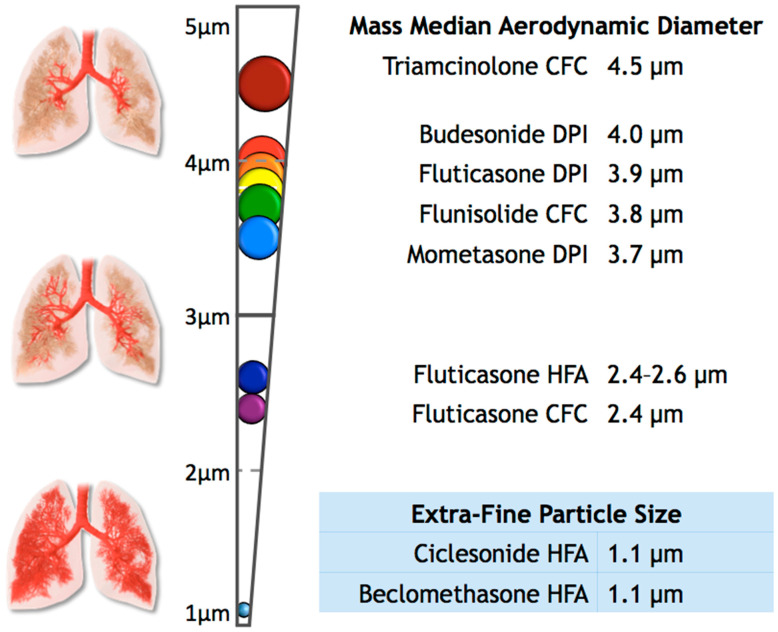
This figure shows the mass median aerodynamic diameter (MMAD) of some common inhaled corticosteroids, as well as the expected distribution of the particle throughout the upper and lower airways [14,23,24,25,26,27,28,29,30,31,32,33,34].

Separately, we explored the effects of the particle size of ICS on the diagnosis of OSA. To perform this analysis, we grouped ICS users into two categories: extra-fine (*n* = 380) and standard size particles (*n* = 14,048). We defined extra-fine-particle ICS as the medications with a mass median aerodynamic diameter (MMAD) of less than 2 µm, which included Ciclesonide HFA and Beclomethasone HFA [14,23,24,25,26]. For the purposes of this study, we grouped all other ICS available in our data as standard-sized particles, ranging in MMAD from 2.4 to 4.5 µm (Figure 1) [14,23,24,25,26,27,28,29,30,31,32,33,34]. We were unable to examine the differences between suspensions and dry powders as well as dosage and potency, since that information was not available in our data. We performed analyses of variance and multivariate logistic regression analyses using these variables, adjusting for sex, BMI group, and race. Statistical analyses were conducted using STATA v14.2 (StataCorp, College Station, TX, USA).

## 3. Results

Table 1 displays the demographic distribution of our initial study cohort as well as the separate cohorts with available ACT and PFT data. The overall cohort consisted of approximately two-thirds females; approximately 80% of the population was Caucasian, and nearly two-thirds were over-weight or obese.

Table 2 shows the number of ICS users across the categories of ICS use, including non-ICS users, standard size particle users, and extra-fine particle size users. We detected some significant differences in asthma control, determined by either ACT scores or FEV_1_ percent predicted values across these categories of ICS particle size (Table 3). When comparing the mean values by unadjusted analysis of variance (ANOVA), there was a significant difference in the ACT scores between each particle-size group (*p* < 0.035); however, when adjusting for sex, race, and BMI group, there was no significant difference between normal and extra-fine users for ACT scores (*p* = 0.077). For FEV_1_ percent predicted scores, the only significant difference was between non-users and normal-particle-size users, and all ICS users vs. non-users (*p* < 0.001, adjusted and unadjusted).

As shown in Table 4, patients with uncontrolled asthma showed increased odds of receiving a diagnosis of OSA, compared to patients with well-controlled asthma as determined by ACT scores (adjusted odds ratio (aOR) 1.60, 95% CI 1.32–1.94; 1380 uncontrolled vs. 3288 controlled) and PFT results (aOR 1.45, 95% CI 1.19–1.77; 1,229 uncontrolled vs. 1,199 controlled). Users of ICS also had increased odds of having a diagnosis of OSA, when compared to non-users of ICS, regardless of asthma control (aOR 1.58, 95% CI 1.47–1.70)). We found increased odds of having OSA for ICS users of standard size particles, compared to non-users (aOR 1.56, 95% CI 1.45–1.69). Increased risk of OSA was not observed when comparing extra-fine particle ICS users to asthmatics that did not use ICS (aOR 1.11, 95% CI 0.78–1.58).

When comparing users of standard size ICS to extra-fine users, unadjusted odds ratios showed increased odds of having a diagnosis of OSA (OR 1.55, 95% CI 1.11–2.16); however, after adjusting for sex, race, and BMI group, the odds were no longer significant (aOR 1.40, 95% CI 0.99–1.58; 13,236 standard size users vs. 380 extra-fine users). Of note, when isolating patients classified as overweight or obese (BMI ≥ 25; *n* = 20,577), standard size particle users did have significantly increased odds of having OSA, compared to extra-fine users (aOR 1.70, 95% CI 1.15–2.50; 9,265 overweight standard size users vs. 241 overweight extra-fine users). Males of all BMI groups who used standard size ICS did not have increased odds of OSA, compared to extra-fine male users (aOR 1.46, 95% CI 0.82–2.57; 4727 male standard size users vs. 131 male extra-fine users). However, overweight and obese males (BMI ≥ 25) who used standard compared to extra-fine size particle ICS did have increased odds for receiving a diagnosis of OSA (aOR 2.45, 95% 1.22–4.93; 3,102 overweight males standard size users vs. 79 overweight male extra-fine users). The odds for females in these circumstances were not significant.

## 4. Discussion

Our results suggest that the use of ICS may be an independent risk factor for the development of OSA, especially in already at-risk patients such as overweight males. The use of ICS may produce local effects on the upper airway dilators that increase the risk of developing OSA, similar to the underlying mechanisms that increase the risk of dysphonia in ICS users [10]. The particle size of ICS likely influences this predisposition for OSA. ICS of larger particle size may produce more localized pharyngeal adverse effects than smaller particle sizes [13,35,36]. In addition, our data suggest that individuals already at risk for developing OSA, such as overweight patients and males [17], may have less risk of developing OSA if they use extra-fine-particle instead of standard-size-particle ICS. Further research is warranted to determine the extent to which extra-fine-particle-size ICS may provide a reasonable alternative for patients with asthma who are at high risk for developing OSA.

As a retrospective cohort study, our data are limited to available variables in the electronic health record. Additionally, asthma and OSA were only defined by ICD-9 or ICD-10 codes, and we did not have data on the dosage, duration of therapy, frequency, adherence, or technique of ICS use. As such, we were unable to detect dose-dependent relationships that prior studies have found, and we were unable to determine if the risk of OSA changes based on the length of time of treatments using different ICS [6]. Although different glucocorticosteroids may differ in both dosage and potency, we were unable to further explore this possible confounder. In addition, we were unable to determine the formulation of the ICS (dry powder or suspensions) for our analysis, and we discarded patients who were prescribed both extra-fine and normal ICS medications during the study period, which may have significant implications in the interpretation of our data. Moreover, we were not able to determine the initial date of diagnosis of OSA or whether patients started using a particular type of ICS prior to receiving a diagnosis of OSA. We assume that the risk increases with prolonged use of ICS, but we cannot confirm this suspicion from our dataset. Lastly, we could not confirm how OSA was diagnosed or treated in our data.

Despite the limitations of our study, our data represent a large cohort of asthmatics with significant results across a demographic distribution reflective of the general US population. Importantly, our data suggest that the particle size of ICS may influence adverse outcomes to include the risk of OSA. Given that ICS are a traditional treatment for chronic asthma, understanding the key differences between treatment options, such as particle size, and potential outcomes may have long-term consequences for the health and quality of life of affected individuals. Furthermore, understanding how these differences of ICS affect individuals depending on their asthma phenotype may provide additional tangible evidence for physicians to seek a wider spectrum of therapies, discouraging the notion of asthma as a “one-size-fits-all” disease. Overall, we believe our results are suggestive of the important differences of the effects of ICS depending on particle size in relation to the development of OSA. Further studies in asthma, especially in obese and male asthmatics, are essential to determine the risks and benefits of the different particle sizes of ICS.

## 5. Conclusions 

Our large retrospective cohort study suggests the use of ICS is an independent risk factor for OSA. Notably, extra-fine particle size ICS do not appear to be associated with an increased risk of OSA. Further prospective studies on this topic are necessary in the future to potentially alter asthma management in those patients at high risk of obstructive sleep apnea.

## Figures and Tables

**Table 1:** Demographic data of the study cohorts.

	Total		With Asthma Control Test Scores		With Pulmonary Function Test Values	
	(*n* = 29,816)		(*n* = 4668)		(*n* = 2428)	
GENDER						
Male	10,803	(36.2%)	1938	(41.5%)	824	(33.9%)
Female	19,013	(63.8%)	2730	(58.5%)	1604	(66.1%)
AGE ^†^						
Mean Age (S.D.)	42.8	(±21.1)	27.9	(±15.5)	55.5	(±18.0)
RACE						
Black or African America	2531	(8.5%)	384	(8.2%)	189	(7.8%)
White or Caucasian	24,026	(80.6%)	3661	(78.4%)	2073	(85.3%)
Other	3260	(10.9%)	623	(13.4%)	166	(6.8%)
Mean BMI (S.D.)	29.8	(±9.0)	27.1	(±8.6)	32.4	(±9.5)
Underweight (BMI < 18)	1470	(5.3%)	512	(11.0%)	44	(1.9%)
Normal (BMI 18–25)	7770	(28.0%)	1726	(37.2%)	479	(20.7%)
Overweight (BMI 25–30)	6593	(30.3%)	1018	(21.9%)	537	(23.2%)
Obese (BMI 30–40)	8393	(30.3%)	1000	(21.5%)	827	(35.8%)
Morbidly Obese (BMI ≥ 40)	3498	(12.6%)	387	(8.3%)	424	(18.4%)
OTHER						
Current Tobacco Users	6250	(21.0%)	694	(14.9%)	598	(24.6%)
Inhaled Corticosteroid Users	14,428	(48.4%)	3355	(71.9%)	1814	(74.7%)

^†^ All patients are age 12 or older. S.D.: Standard deviation BMI: Body Mass Index.

**Table 2:** Number of ICS users by particle size and asthma control categories.

	ICS Non-Users	ICS Users		
		Normal Particles	Extra-fine Particles	Total ICS Users
Initial Cohort	15,388	14,048	380	14,428
ACT Control Categories ^†^	1313	2881	118	3355
Uncontrolled	181	998	34	1199
Well Controlled	1132	1883	84	2156
PFT Control Categories ^#^	547	1614	27	1814
Uncontrolled	210	891	8	989
Well Controlled	337	723	19	825

^†^ An ACT score of less than 20 was established to indicate uncontrolled asthma. ^#^ Forced expiratory volume in 1 second (FEV_1_) percent that predicted values of 80 or less was used to indicate uncontrolled asthma. ICS: inhaled corticosteroids. ACT: asthma control test. PFT: pulmonary function tests.

**Table 3:** Mean ACT and PFT values by ICS particle size.

	Mean Value *	Standard Deviation	Number of Patients
ACT Scores (Total)	20.87	4.44	4668
All ICS users	20.19	4.67	3355
Normal particle size	20.30	4.62	2881
Extra-fine particle size	21.21	3.52	118
Non-users	22.61	3.17	1313
FEV_1_% Predicted (Total)	78.01	20.76	2361
All ICS users	76.12	20.91	1814
Normal particle size	75.95	21.00	1614
Extra-fine particle size	81.74	20.56	27
Non-users	83.78	19.36	547

* When comparing these means by unadjusted analysis of variance (ANOVA), there was a significant difference in ACT scores between each particle-size group (*p* < 0.035); however, when adjusting for sex, race, and BMI group, there was no significant difference between normal and extra-fine users for ACT scores (*p* = 0.077). For FEV_1_ percent predicted scores, the only significant difference was between non-users and normal-particle-size users, and all ICS users and non-users (*p* < 0.001, adjusted and unadjusted). Notably, for analysis, three distinct ANOVA’s were performed comparing two groups at a time.

**Table 4:** Odds for having a diagnosis of OSA.

	Unadjusted Odds Ratio	95% CI	Adjusted Odds Ratio *	95% CI
Uncontrolled vs. Controlled Asthma:				
As determined by ACT score	2.00	1.67–2.40	1.60	1.32–1.94
As determined by PFT results	1.66	1.39–1.99	1.45	1.19–1.77
ICS Users and Categories:				
ICS users (any particle size) vs. non-users	1.69	1.58–1.81	1.58	1.47–1.70
Normal size particle ICS users vs. non-users	1.62	1.51–1.74	1.56	1.45–1.69
Extra-fine particle ICS users vs. non-users	1.05	0.75–1.46	1.11	0.78–1.58
Compared to Extra-Fine Size ICS Users:				
Normal size ICS users	1.55	1.11–2.16	1.40	0.99–1.98
Normal size ICS with BMI ≥25 only	1.70	1.16–2.51	1.70 ^#^	1.15–2.50
Normal size ICS, males only	1.68	0.97–2.88	1.46 ^+^	0.82–2.57
Normal size ICS, males with BMI ≥25 only	2.44	1.21–4.90	2.45 ^†^	1.22–4.93

* Adjusted for for sex, race, smoking and BMI group. # Adjusted for sex and race. ^+^ Adjusted for race and BMI group. ^†^ Adjusted for race. OSA: obstructive sleep apnea CI: confidence interval BMI: body mass index.
